# Metastatic Undifferentiated Osteoclast-Like Giant Cell Pancreatic Carcinoma

**DOI:** 10.7759/cureus.27586

**Published:** 2022-08-01

**Authors:** Mehdi B Swaid, Erin Vitale, Nieem Alatassi, Hassaan Siddiqui, Habeeb Yazdani

**Affiliations:** 1 Internal Medicine, Michigan State University College of Osteopathic Medicine, East Lansing, USA; 2 Internal Medicine, Ascension St. John Hospital, Warren, USA

**Keywords:** gastrointestinal cancer, metastatic, giant cell tumors, gastrointestinal bleed, mucinous cystic neoplasms, pancreatic cancer, osteoclast-like giant cell pancreatic carcinoma, undifferentiated osteoclast-like giant cell pancreatic carcinoma

## Abstract

Undifferentiated osteoclast-like giant cell pancreatic carcinoma (UC-OGC) is a rare pancreatic carcinoma that is composed of osteoclast-like giant cells among other cells and is hardly described in literature due to its infrequent presentation. With that, a rare symptom of pancreatic cancers is upper gastrointestinal (GI) bleeding. We report a 76-year-old African American male who presented with one episode of bloody emesis associated with intermittent episodes of severe abdominal pain and a 25 lbs of unintentional weight loss secondary to metastatic UC-OGC. The patient was stabilized and referred to an oncologist for further treatment. We present this case to add to the existing literature on UC-OGC of the pancreas.

## Introduction

Mucinous cystic neoplasms (MCNs) are rare lesions of the tail of the pancreas, accounting for 2%-5% of all exocrine pancreatic lesions [1]. They are typically formed in the tail of the pancreas and are usually noninvasive; however, they can undergo malignant changes. When invasive, MCNs can transform into a variety of other types of malignant lesions but less commonly develop into undifferentiated osteoclast-like giant cell tumors [2].

Histologically, it is suggested that undifferentiated osteoclast-like giant cell pancreatic carcinoma (UC-OGC) comes from both epithelial and mesenchymal origin, with positive immunohistochemical staining for CEA and keratin favoring epithelial origin and positive CD68 and vimentin favoring mesenchymal derivation. Compared to its counterpart, pleomorphic giant cell tumor and adenocarcinoma, UC-OGC has a better prognosis because it is histologically less aggressive with slow metastasis. The survival rate can vary from person to person from four months to 10 years. It has a similar outcome to ductal pancreatic cancer, however, with a median patient survival after diagnosis of 11 months. This is most likely due to it being diagnosed late in the cancer process [3,4]. The following case demonstrates a 76-year-old male with a rare presentation of rare cancer.

## Case presentation

We report on a 76-year-old African American male with no relevant medical history throughout his life who was brought to the emergency department by his wife for a syncopal episode. For the past two months, he has been having intermediate episodes of severe upper abdominal pain, nausea, dry heaves, non-bloody emesis, early satiety, increased frequency of stooling, and a 25-lb unintentional weight loss over a two-month period. At the time, he denied diarrhea, melena, and hematochezia. The abdominal examination was positive for tenderness to palpation of both upper quadrants, and the rest of the physical examination was unremarkable. Blood work and imaging were ordered on admission, and the laboratory results are shown in Table 1.

**Table 1:** Pertinent laboratory results during the first admission L: laboratory value below the reference range, H: laboratory value above the reference range, TIBC: total iron-binding capacity

Collected parameter	Value	Reference range
WBC	7.06	4.50-10 × 10^3^/uL
RBC	2.79 L	4.40-5.60 × 10^6^/uL
HBG	8.2 L	13-17 g/dL
HCT	24.5 L	39.6%-50%
MCV	87.8	80-97 fL
MCH	29.4	27-33.9 pg
MCHC	33.5	33.2-35.4 g/dL
RDW	14.4	11.5%-14.5%
PLT	190	140-440 × 10^3^/uL
Iron	29 L	65-175 ug/dL
TIBC	153 L	228-460 ug/dL
Iron saturation	18.95	15%-50%
Na^+^	141	135-147 mmol/L
K^+^	2.90 L	3.50-5.30 mmol/L
Cl^-^	104	96-112 mmol/L
BUN	7	7-20 mg/dL
CRE	0.730	0.5550-1.300 mg/dL
BUN/CR	9.59 L	12-20
Ca^2+^	8.80	8.20-10.60 mg/dL
Direct bilirubin	1.02 H	0-0.40 mg/dL
Total bilirubin	2.10 H	0.20-1 mg/dL
Indirect bilirubin	1.08 H	0-1 mg/dL
Alkaline phosphatase	107	41-126 U/L
ALT	70 H	10-49 U/L
AST	68 H	14-35 U/L

A computed tomography (CT) scan of the abdomen and pelvis with contrast revealed several pancreatic lesions along with ductal dilation within the tail of the pancreas, dilation of the common bile duct (CBD), and intrahepatic biliary ducts. One left kidney lesion and multiple hepatic lesions were also visualized. This prompted an endoscopic retrograde cholangiopancreatography (ERCP) with cold snare removal of a protruding bile duct stone, sphincterotomy, brushing of the CBD for malignancy, and placement of a metal stent with adequate drainage. Cytology report from CBD brushing revealed no malignant cells. A magnetic resonance imaging (MRI) with and without contrast revealed an 8.2 × 5.1 cm expansile pancreatic body/tail mass with pancreatic ductal dilation, a 3.1 × 2.9 cm pancreatic head mass, a 2.1 × 2 cm left kidney mass, and multiple hepatic lesions consistent with metastatic disease. Interventional radiology was consulted, and the patient underwent an ultrasound-guided biopsy of liver lesions. Tumor cells were positive for CD68, CD163, and vimentin histiocytic markers in keeping with monocytic differentiation of osteoclastic giant cells. KRAS, ARID1A, CDKN2A, DKM6A, NF1, and TP53 mutations were observed in the tumor cells. Histochemical and morphologic features were consistent with osteoclast-like giant cell tumors of the pancreas. Once stabilized, the patient was discharged with instructions to follow up with an oncologist.

**Figure 1:**
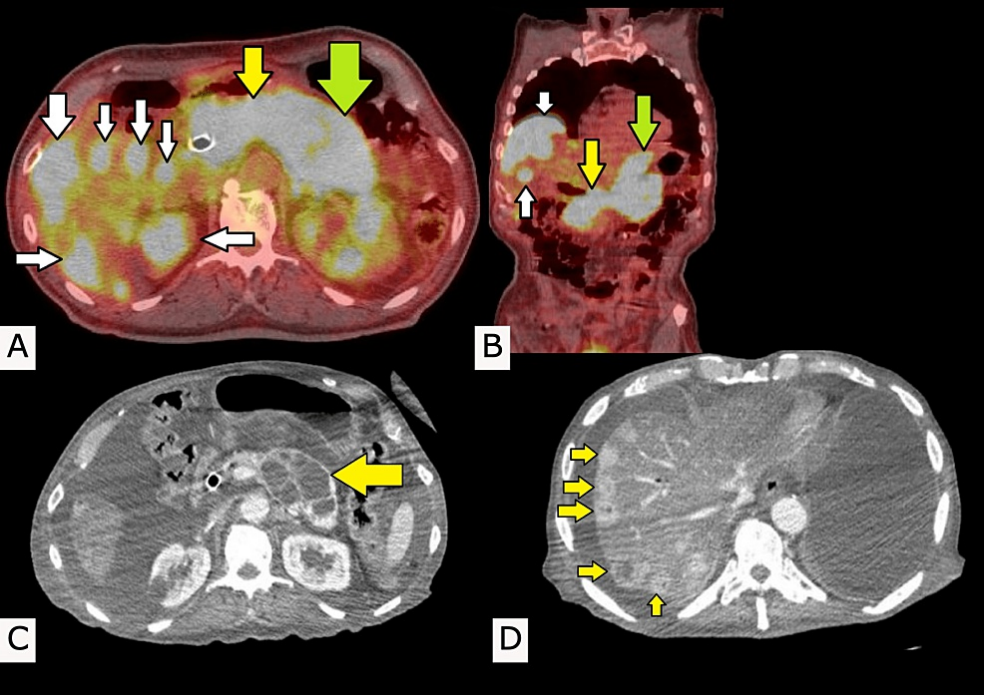
Positron emission tomography (PET) scans and computed tomography (CT) scans (A) Axial and (B) coronal PET scans displaying a large mass involving the body (yellow arrows) and tail (green arrows) of the pancreas with innumerable masses throughout the liver (white arrows). These lesions with considerate metabolism are indicated by hyperlucent (bright white) areas on the scan. (C) Multicystic enlargement of the body and tail of the pancreas (yellow arrow). (D) Multiple enhanced lesions of the liver, some of which are cystic or necrotic in nature (yellow arrows).

One month later, he then presented to the emergency department for two syncopal episodes and was accompanied by hematemesis with a hemoglobin of 6.8 gm/dL and reticulocytes at 3.16%. The patient was transfused one unit of blood for low hemoglobin and underwent esophagogastroduodenoscopy, which was unremarkable. The upper gastrointestinal (GI) bleed was presumed to be due to the pancreatic masses. Furthermore, a positron emission tomography-computed tomography (PET-CT) was done, and it revealed an innumerable amount of lesions throughout the liver along with abnormal metabolism with the standardized uptake values (SUVs) in the 14-18 range. There was also a large mass involving the body and tail of the pancreas with considerable metabolism, with a maximum SUV of about 18. Additionally, a bile duct stent was seen in the distal common bile duct region, placed a few weeks prior (Figure 1A, 1B). The patient was eventually deemed stable by all teams and was discharged home with instructions to follow up with oncology for further treatment.

After his discharge, the patient further underwent treatment with the chemotherapy regimen of Folfirinox. During his treatment at our facilities, CT images with contrast were taken (Figure 1C, 1D). The radiologist reported diffuse hepatic steatosis with numerous enhancing lesions, many of which have central cystic/necrotic components scattered throughout the liver. There is pneumobilia with a common bile duct stent in place. Multi-lobular cystic enlargement involves the body and tail of the pancreas, which contain multiple thickened internal septations. There was a hypoattenuating lesion in the left kidney. He continued his regime for six months with lesions mildly decreasing in size, until he transitioned to hospice care.

## Discussion

UC-OGC is difficult to diagnose due to its similar presentation to pancreatic ductal adenocarcinoma. Additionally, UC-OGC can be described as “pure” or as a “mixed” carcinoma associated with other types of pancreatic cancers. The genetic pathways of pure and mixed carcinoma are described in the literature. Some differentiating signs between ductal adenocarcinoma and UC-OGC are on imaging; typically, UC-OGC is larger and has cystic components compared to ductal adenocarcinoma. UC-OGC can produce an intraductal polypoid mass causing dilation of the bile and pancreatic duct, leading to hyperbilirubinemia and/or jaundice and associated symptoms, similar to our case. Also, calcification, necrotic areas, venous tumor thrombosis, and hemorrhage have been described in these tumors as well. However, the best way to diagnose UC-OGC is through biopsy and immunohistochemical staining [5].

On histology, UC-OGC appears as benign giant cell tumors of bone that contain a combination of osteoclast-like multinucleated and mononuclear cells. Histologically, it is suggested that UC-OGC comes from both epithelial and mesenchymal origin, staining positive for CEA and keratin favoring epithelial origin and positive for CD68 and vimentin favoring mesenchymal derivation, and can or cannot contain mutations against p53. However, it has a similar outcome to common ductal pancreatic cancer, with a median patient survival after diagnosis of 12 months [3-6]. In our patient’s case, tumor cells were positive for KRAS, ARID1A, CDKN2A, DKM6A, NF1, and TP53 mutations. Other literature describes mutations in KRAS, TP53, CDKN2A, and SMAD4, but as far as we know, none describe DKM6A and NF1 mutations correlating with UC-OGC [5].

There is no standardized treatment for UC-OGC because of how rare the occurrence of this disease is. Thus, chemotherapy and/or radiation therapy for cancer is not well documented [5,7]. However, some literature suggests using the standardized treatment regime for ductal pancreatic carcinoma because UC-OGC is a variant. The first-line treatment for ductal pancreatic carcinoma is surgical resection with or without conjunctive chemotherapy and/or radiation therapy. A few articles also proposed using monoclonal antibodies against PD-1 and PD-L1 due to their effectiveness against a wide variety of cancers, including giant cell cancers in other organs [5,8-13]. Finally, when taking the approach to treating this and other rare cancers, the providers need to have flexible thinking and creative use of their knowledge of the pathophysiology of these cancers to treat them properly. 

Gastrointestinal bleeding is a rare complication of pancreatic cancer. Only 2.6% of patients with pancreatic cancer experience an episode of upper GI bleeding [14]. The diagnosis can be established using endoscopic or surgical methods, although conventional endoscopic evaluation has been unsuccessful in some cases, including the case in point. Physicians should be aware that the severity of the bleeding in these patients can vary from occult bleeding to severe hemorrhagic bleeding, leading to hypovolemic shock. Treatment options include endoscopic hemostasis, arterial embolization, and surgery. Failure to achieve hemostasis results in an extremely poor prognosis [14].

## Conclusions

We present this case to add to the existing literature on UC-OGC of the pancreas. Due to the rarity of this condition, it renders the possibility of cohort studies unlikely, leaving case reports and meta-analyses as the majority of literature on this topic. With that, long-term follow-up along with thorough and detailed history-taking of these patients is important to guide management and understand the prognosis of this cancer. In the existing literature, the number one treatment option is surgery. However, in this case, due to metastasis, chemoradiation or immunotherapy may be considered as initial management or conjunctive treatment with surgery. Finally, to our best knowledge, no case of UC-OGC described in the literature presented with an upper gastrointestinal bleed.
